# FINANCIAL TRANSFERS BETWEEN OLDER FILIPINOS AND THEIR ADULT CHILDREN: THE ROLE OF EDUCATION AND PUBLIC PENSIONS

**DOI:** 10.1093/geroni/igae098.3606

**Published:** 2024-12-31

**Authors:** Kent Jason Cheng

**Affiliations:** The Pennsylvania State University, University Park, Pennsylvania, United States

## Abstract

**Objectives:**

The Philippines is expected to transition to an aging society in less than two decades. Provision of resources and safety nets largely befalls family members since government support is limited. This study examines how education and public pensions shape older Filipinos’ financial transfers to and from their adult children.

**Methods:**

Data on Filipinos aged 60+ with at least one living child came from the 2018 Longitudinal Study of Ageing and Health in the Philippines (n=5,645). Logistic regressions interacting educational attainment and public pension receipt (no pensions, have contribution-based pensions, have social pensions, have both) were ran, controlling for relevant sociodemographic factors, average characteristics of children, and living arrangements. Predicted probabilities were generated and pairwise comparisons were conducted.

**Results:**

Higher educational attainment and having contribution-based public pensions were associated with higher (decreased) odds of giving (receiving) transfers. Interactive models suggest a modest moderating effect of education and public pension types on giving and receiving financial transfers. Within education level, public pension receivers, especially those with contribution-based pensions, tend to have the highest probability of transferring to children and lowest probability of being exclusively on the receiving end of money transfers.

**Discussion:**

Public pension receivers being more likely to give downward financial transfer across all educational levels suggests that public transfers for older adults benefit both the intended beneficiaries and their descendants. Additionally, older parents having contribution-based pensions as an income source spares their adult children from providing upward transfers. Results support crowding in – that public transfers facilitate private transfers.

